# Double Fano Resonance and Independent Regulation Characteristics in a Rectangular-like Nanotetramer Metasurface Structure

**DOI:** 10.3390/nano12193479

**Published:** 2022-10-05

**Authors:** Zhidong Zhang, Qingchao Zhang, Bo Li, Junbin Zang, Xiyuan Cao, Xiaolong Zhao, Chenyang Xue

**Affiliations:** 1 Key Laboratory of Instrumentation Science and Dynamic Measurement of Ministry of Education, North University of China, Taiyuan 030051, China; 2 School of Software, North University of China, Taiyuan 030051, China; 3 School of Electrical and Control Engineering, North University of China, Taiyuan 030051, China

**Keywords:** surface plasmon resonance, double fano resonance, plasmonic metasurface, finite element method

## Abstract

Fano resonance, which is based on a plasmonic metasurface, has many potential applications in various fields, such as biochemical sensors, slow light effect, and integrated optical circuits. In this study, a rectangular-like nanotetramer metasurface structure composed of four round-head nanorods was designed. The transmission spectrum, surface charge, and electrical field distributions of the proposed structure were simulated using the finite element method. A double Fano resonance profile was observed in the transmission spectrum. One of the Fano resonances was caused by the symmetry breaking and plasmon hybridization between the horizontal double rods, whereas the other resonance was due to the plasmonic modes’ hybridization among four nanorods. These resonances could be independently tuned because of different formation mechanisms. The number of Fano resonances could be adjusted by changing the coupling distance between the horizontal and vertical rods. The results contributed to designing the highly sensitive sensors based on the plasmonic metasurface.

## 1. Introduction

The surface-free electrons of noble metal nanoparticles are driven by the electrical field of incident light, resulting in the generation of surface plasmon resonance (SPR) when the frequency of the free electron oscillation is equal to the incident light [1,2,3]. SPR can limit the electromagnetic field to subwavelength scale and enhance a localized field at a nano scale [4]. This property is widely used in surface-enhanced Raman scattering [5,6], surface-enhanced fluorescence [7], and sensors [8,9,10].

Fano resonance, as a special plasmon resonance, has been recently observed in plasmonic metasurface structures, which results from the plasmonic mode hybrid [11,12]. Researchers have found that the plasmonic mode hybrid is achieved through the bonding and antibonding modes [13,14]. The bonding mode can directly interact with incident light because of its large damping radiation, which leads to resonance broadening; thus, it is also called the bright mode. The antibonding mode cannot directly interact with incident light because of its small damping radiation, which leads to resonance shrinking; thus, it is also called the dark mode [15,16]. Compared with the conventional plasmon resonance, Fano resonance has a series of excellent properties, such as sharp asymmetry line shape, strong electromagnetic enhancement, and high sensitivity sensing, thereby being widely applied to biosensors [17,18,19,20], slow light effect [21,22,23], integrated optical circuits [24], and enhanced nonlinear optics [25,26,27]. In addition, the double Fano resonance phenomenon is observed in the transmission spectra of polymer metasurfaces due to the near-field interaction between polymers [28]. It has also been used to design the switch [29,30]. For several special applications, the double Fano resonance should be independently tunable [31]. However, the Fano resonance peaks are closely related to each other in previous double Fano resonance systems, making it difficult to regulate independently.

Therefore, the different hybrid ways were combined to design an independent-tunable double Fano resonance plasmonic system. A plasmonic rectangular nanotetramer structure is proposed to achieve the double Fano resonance. The transmission spectra, surface charge and electrical field distributions of this structure were simulated using the finite element method. The physical mechanism of Fano resonance was investigated by comparing the transmission spectra of the related structures and analyzing the corresponding charge and electrical field distributions. Meanwhile, the influence of structural parameters on the Fano resonance was also investigated. These results provided a substantial theoretical basis for designing highly sensitive sensors.

## 2. Structure and Method

**Figure 1:**
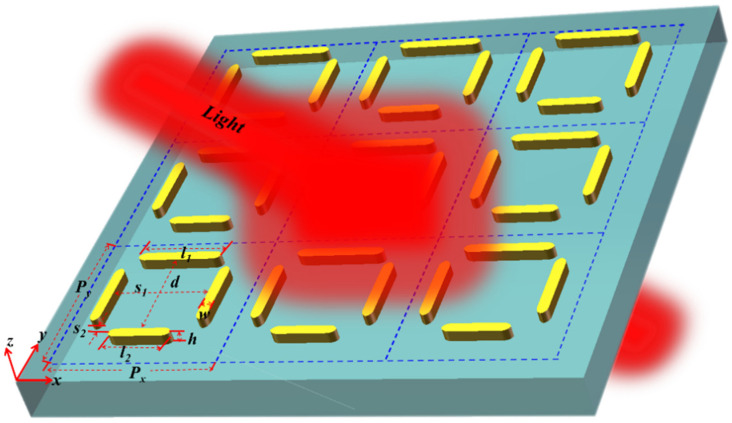
Periodic array diagram of rectangular-like nanotetramer metasurface structure.

Figure 1 schematically depicts the periodic array of the rectangular nanotetramer structure and the top view of its unit cell. The unit cell consists of four round-head gold nanorods, which are located on the SiO_2_ dielectric layer with a thickness of 200 nm. All gold nanorods have the same thickness of *h* = 40 nm and width of *w* = 20 nm. The length of the horizontal top nanorod is equal to the vertical nanorods (*l*_1_ = 260 nm), and the length of the horizontal bottom nanorod is *l*_2_. The distance of horizontal and vertical rods is *d* and *s*_1_, respectively. The vertical distance between the horizontal bottom nanorod and the vertical nanorods is *s*_2_. The unit cells are arranged with periods of *P_x_* = 400 nm and *P_y_* = 600 nm in the *x* and the *y* directions.

In this study, all numerical simulations are achieved by the wave optics module of the COMSOL Multiphysics^®^ 5.2a software. The incident light is along the *z* direction, whereas the polarization is along the *x* direction. In addition, the perfectly matched layer boundary conditions are used for the *z* directions, whereas the periodic boundary conditions are used for the other directions.

## 3. Results and Discussion

**Figure 2:**
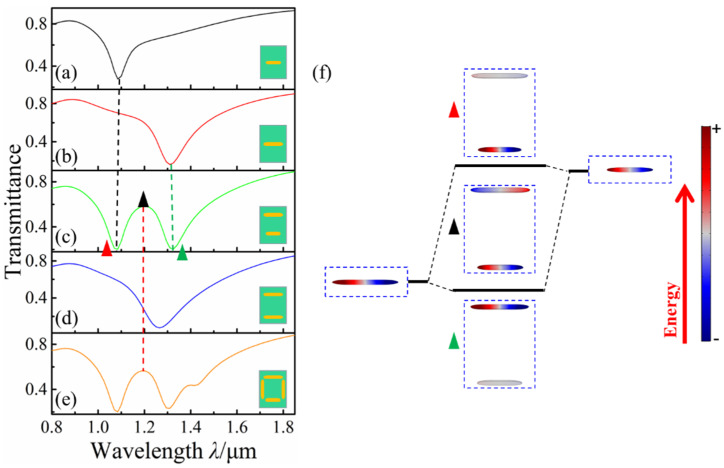
(**a**–**e**) Transmission spectra of the horizontal bottom nanorod, horizontal top nanorod, asymmetric horizontal double nanorods, and symmetric horizontal double nanorods and the rectangular nanotetramer, respectively; (**f**) Hybrid scheme of asymmetric horizontal double nanorods.

Figure 2a,e show the transmission spectra of the horizontal bottom nanorod with *l*_2_ = 200 nm, the horizontal top nanorod with *l*_1_ = 260 nm, the asymmetric horizontal double nanorods with *l*_2_ = 200 nm and *l*_1_ = 260 nm, the symmetric horizontal double nanorods with *l*_1_ = *l*_2_ = 200 nm, and the rectangular nanotetramer structure (*l*_1_ = 260 nm, *l*_2_ = 200 nm, *d* = 320 nm, *s*_1_ = 300 nm, *s*_2_ = 40 nm). Compared with Figure 2a,c shows two transmission dips and a transmission peak. Among them, each transmission dip corresponds to the horizontal top and bottom nanorods, respectively. The transmission peak disappears when the length of the horizontal double rods is equal, as shown in Figure 2d. Therefore, the asymmetrical profile composed of two transmission dips and a transmission peak is considered Fano resonance, which comes from the breaking symmetry of the horizontal double nanorods. To explain the physical mechanism of this process, the charge distributions and hybrid scheme of the horizontal top nanorod, the horizontal bottom nanorod, and asymmetric horizontal double nanorods are simulated and shown in Figure 2f. The solid black lines of different heights represent high and low energy in Figure 2f. The transmission dips of the horizontal top and bottom nanorods are caused by dipole oscillations, and the dipole of the horizontal top and bottom nanorod located in low and high energy state, respectively. The dipole vibration at the double transmission dips of the asymmetric horizontal double nanorods from the horizontal bottom and top nanorods, respectively. However, the two transmission dips of the asymmetric horizontal double nanorods are slightly shifted to shorter and longer wavelengths relative to the individual nanorods, respectively. At the same time, we also found that the antiphase charge oscillation is formed at the transmission peak of the asymmetric horizontal double nanorods as a result of the near-field interaction between the double nanorod dipole oscillations. Figure 2e shows the transmission spectra for the rectangular nanotetramer. The profiles of double Fano resonance are observed, and the resonance at the short wavelength corresponds with the asymmetric horizontal double nanorods, indicating that this resonance is due to the breaking symmetry of the horizontal double nanorods in the rectangular nanotetramer.

**Figure 3:**
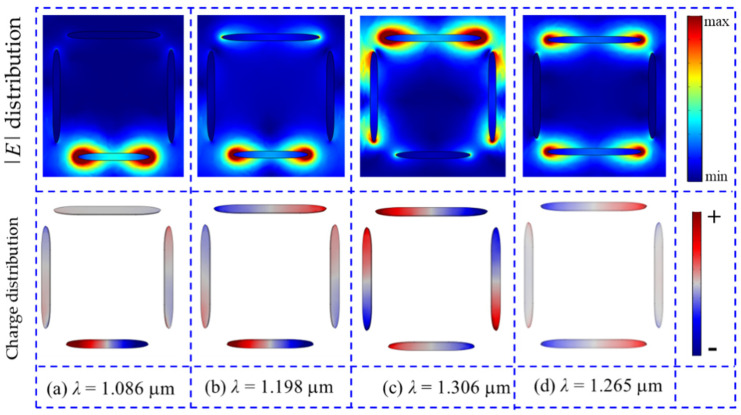
The electrical field |*E*| and surface charge distributions corresponding to the transmission peak (*λ* = 1.198 μm) and transmission dips (*λ* = 1.086 μm, *λ* = 1.306 μm) of the asymmetry horizontal top and bottom nanorods. The electrical field |*E*| and surface charge distributions corresponding to the transmission dip (*λ* = 1.265 μm) of the symmetry horizontal top and bottom nanorods.

To further verify that the Fano resonance of the short wavelength is formed by the breaking symmetry of the horizontal double nanorods in the rectangular nanotetramer, the distributions of the normalized electrical field and surface charge at the transmission peak and the two transmission dips are simulated and shown in Figure 3. For Figure 3a *λ* = 1.086 μm, the electrical field radiation is very weak except for the ends of the horizontal bottom rod, and the charge’s distribution shows a dipole oscillation mode. Therefore, a super-radiation dipole mode is formed in the horizontal bottom nanorod. For Figure 3b *λ* = 1.198 μm, the electrical field is mainly distributed near the two ends of the double asymmetric horizontal rods and the charge, which emerges in antiphase distribution and is caused by the near-field interaction between the dipole modes of the horizontal double rods. For Figure 3c *λ* = 1.306 μm, the electrical field can be observed near the four nanorods, and the strongest electrical field radiation occurs at the ends of the horizontal top nanorod. As shown in Figure 3c, we see that the near-field interaction is weak among the vertical double nanorods and horizontal bottom nanorod, but the interaction is stong among the horizontal top nanorod and the vertical double nanorods. From the charge distribution, we see that the charge has the opposite distribution at the vertical double nanorods, so it is antiphase dipole charge distribution of the vertical double nanorods. When the asymmetric nanotetramer is incident by the light of *λ* = 1.306 μm, which corresponds to the resonance dip of the single horizontal top nanorod. The enhanced electrical fields, which are caused by the dipole vibration, occurred at two ends of the horizontal top nanorod. The inductive electrical fields around two ends of the vertical double nanorods stimulated by these enhanced electrical fields, which lead the dipole vibration, occur at the vertical double nanorods. Then, the inductive electrical fields of the horizontal bottom nanorod are inducted by the enhanced electrical fields around the vertical double nanorods. But the horizontal bottom nanorod and vertical double nanorods have a weak coupling due to the fact that the distance between them is larger. For the symmetric structure, the electrical |*E*| and charge distributions at the transmission dip (*λ* = 1.265 μm) are shown in Figure 3d. We found that the dipole mode is observed distinctly in the horizontal top and bottom nanorods. The near-field coupling with the horizontal double nanorods occur at the two ends of the vertical nanorods.

**Figure 4:**
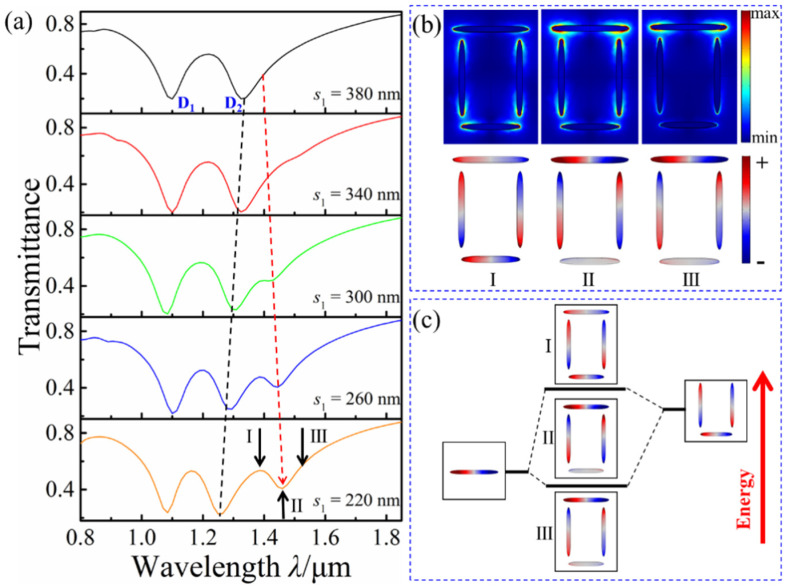
(**a**) Transmission spectra with the different *s*_1_; (**b**) charge and electrical field distributions at positions I, II, and III; (**c**) hybrid mode scheme between the horizontal top nanorod and the remaining nanorods.

As for the Fano resonance at the longer wavelength, the influence of coupling strength on it is firstly investigated by changing the coupling distance. The transmission spectra with a different *s*_1_ is shown in Figure 4a, with *l*_1_ = 260 nm, *l*_2_ = 200 nm, *d* = 320 nm, and *s*_2_ = 40 nm. During the decrease in *s*_1_, the Fano resonance profile at the long wavelength becomes increasingly obvious and the transmission dip D_2_ has a slight blue-shift, but D_2_ no shift. To study the formation mechanism of the new Fano resonance, the distributions of the charge and the electric field at different incident wavelengths (I: *λ* = 1.385 μm, II: *λ* = 1.462 μm, and III: *λ* = 1.518 μm) in the transmission spectrum with *s*_1_ = 220 nm are simulated, as shown in Figure 4b. For *λ* = 1.385 μm, the electrical field is mainly distributed at the two ends of the horizontal bottom nanorod and the vertical nanorods. The horizontal and vertical double nanorods’ charges oscillate in the same phase and antiphase, respectively. This finding reveals the excitation of the bonding mode among the four rods. For *λ* = 1.518 μm, the electrical field is mainly distributed at the two ends of the horizontal top nanorod. The charge oscillation is similar to *λ* = 1.385 μm, which reveals the excitation of the antibonding mode. For *λ* = 1.462 μm, the electrical field is distributed at the two ends of each nanorod and at the gaps between these nanorods. The horizontal and vertical double nanorods’ charges show antiphase oscillation, which is considered a transition mode formed by the interaction between the bonding and antibonding modes. The radiation loss of the electrical field is enhanced in the transition mode. Figure 4c shows the hybridization scheme of the horizontal top nanorod interacting with the remaining nanorods. The transmission dip gradually becomes obvious with the decrease in *s*_1_ due to the enhanced interaction between the bonding and antibonding modes.

**Figure 5:**
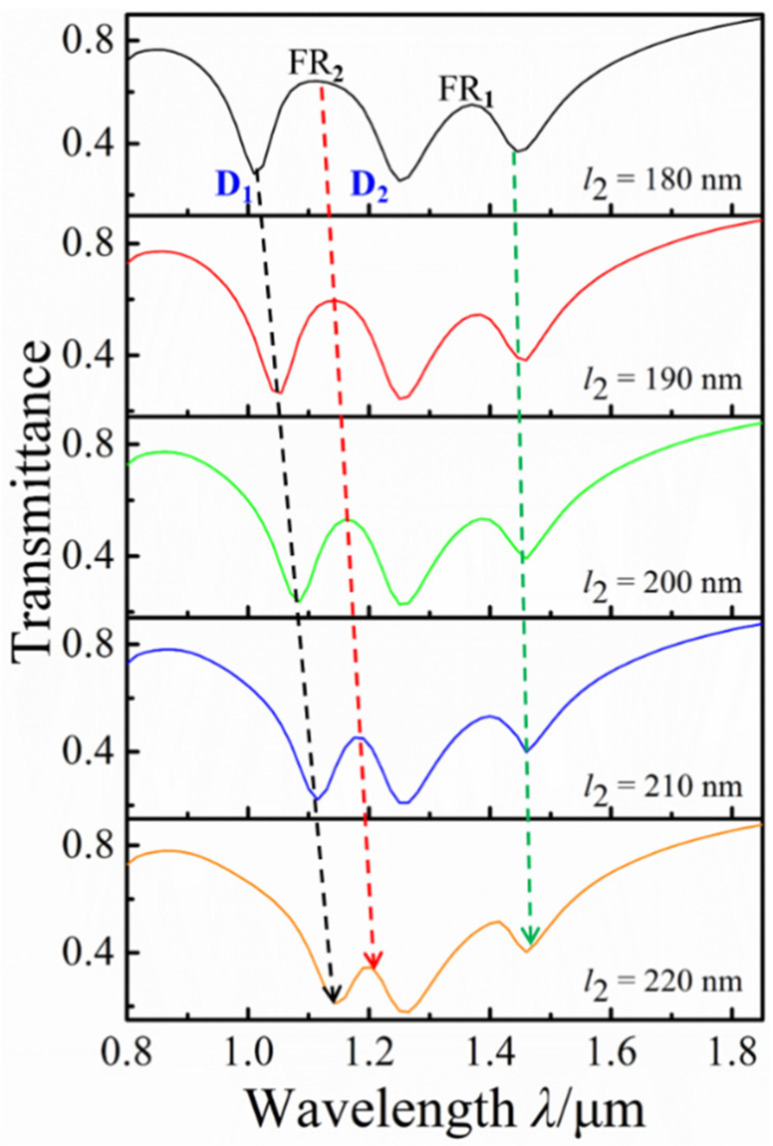
Transmission spectra with different horizontal bottom nanorod lengths *l*_2_.

Fano resonance, as a weak interaction, strongly depends on structural parameters. The effect of the horizontal short rod *l*_2_ on the Fano resonance of the proposed structure is investigated by increasing *l*_2_ from *l*_2_ = 180 nm to *l*_2_ = 220 nm, with *l*_1_ = 260 nm, *d* = 320 nm, *s*_1_ = 220 nm, and *s*_2_ = 40 nm. Figure 5 shows that the transmission spectra under different horizontal bottom nanorod lengths *l*_2_. With the increase in *l*_2_, the transmission dip D_1_ blue-shifts and the modulation depth of the FR_2_ is gradually reduced, which is due to the reduction of symmetrical damage between the horizontal double rods. However, the FR_1_ hardly have any changes with the increase in *l*_2_. The change of *l*_2_ mainly affects the FR_2_, and we could independently tune the FR_2_ through this change.

**Figure 6:**
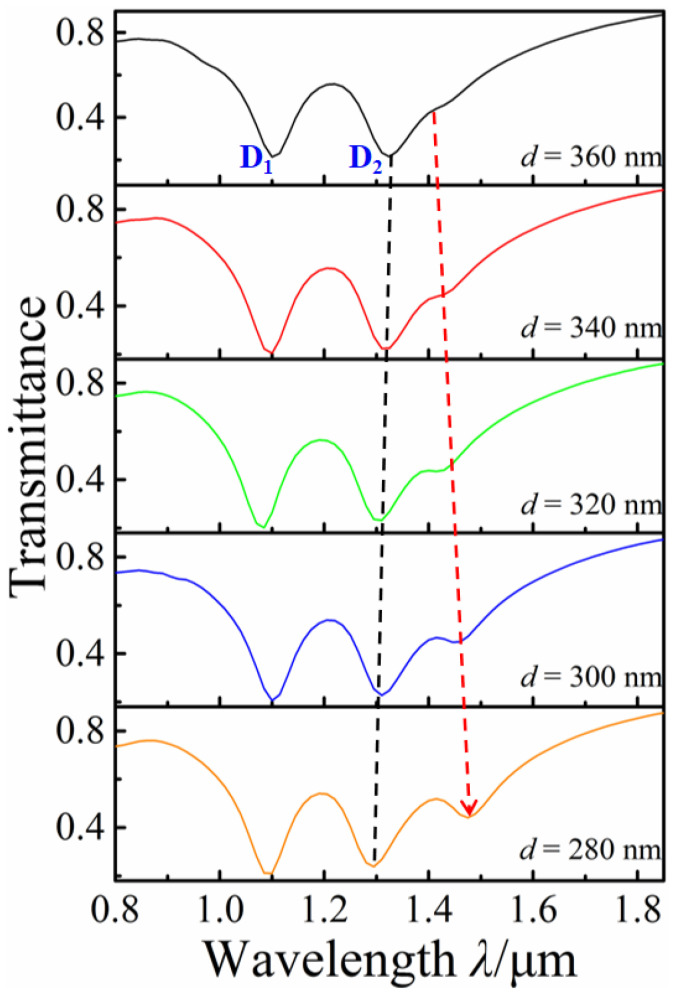
Transmission spectra with the different horizontal double nanorod distance *d*.

The influences of the distance *d* between the horizontal double nanorods on the Fano resonance are also investigated by decreasing *d* from *d* = 360 nm to *d* = 280 nm, with *l*_1_ = 260 nm, *l*_2_ = 200 nm, *s*_1_ = 300 nm, and *s*_2_ = 40 nm, as shown in Figure 6. With the decrease in *d*, the transmission dip D_2_ slightly blue-shifts and a distinct transmission dip is gradually generated, resulting in the increase in the depth of FR_1_ modulation. It is due to the gradual enhancement of the interaction between the plasmonic bonding and antibonding modes, which are formed by the horizontal top nanorods and the remaining nanorods. The change of *d* mainly affects the FR_1_, and we can independently tune the FR_1_ through this change.

**Figure 7:**
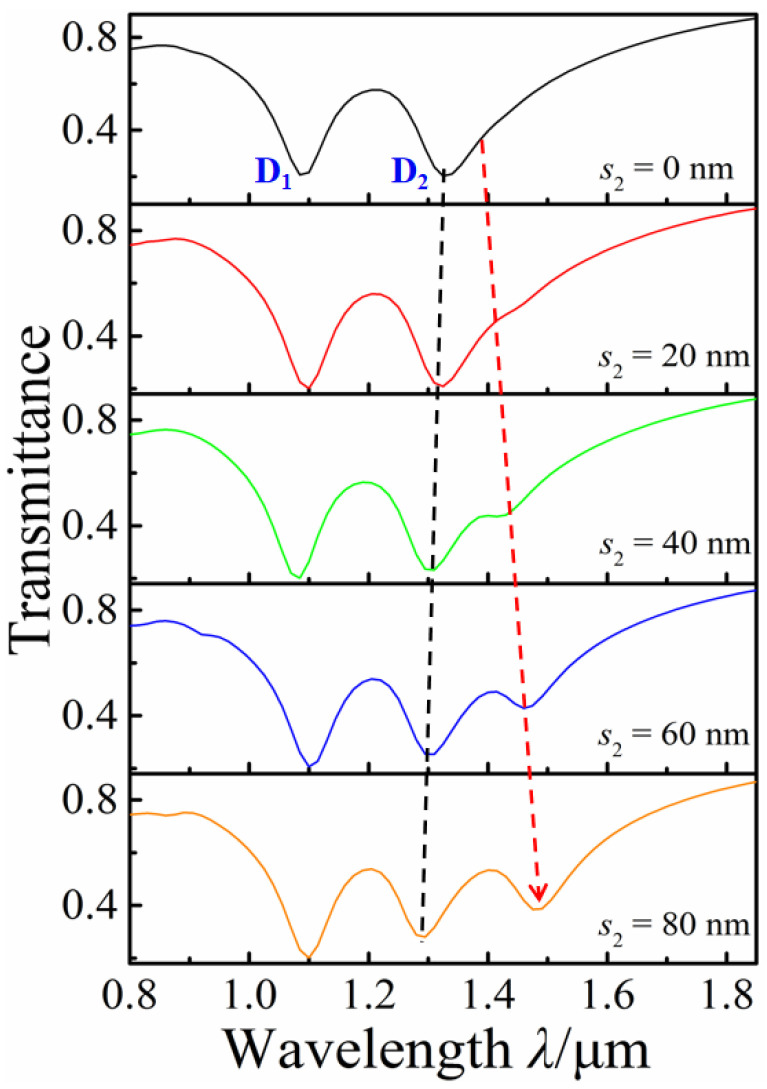
Transmission spectra of *s*_2_ increasing from 0 nm to 80 nm.

The adjustment of FR_1_ can also be achieved by moving the vertical double nanorods up and down. Figure 7 shows the transmission spectra of *s*_2_ increasing from 0 nm to 80 nm, with *l*_1_ = 260 nm, *l*_2_ = 200 nm, *s*_1_ = 320 nm, and *d* = 320 nm. We found that the transmission dip D_2_ slightly blue-shifts, and a distinct transmission dip is gradually generated with the increase in *s*_2_. Its modulation mechanism for FR_1_ is similar to changing the distance between the horizontal double rods.

## 4. Conclusions

A rectangular nanotetramer structure is proposed and investigated via the finite element method. The transmission spectra, surface charge and al field distributions of the related structures are investigated and analyzed. The single and double Fano resonance profiles can be observed in the transmission spectra by changing the structural parameters. The generation mechanisms of two Fano resonances are different. One is caused by the symmetry breaking and plasmon hybridization between the horizontal double nanorods in the rectangular nanotetramer, and the other is due to the plasmonic modes hybridization among four nanorods. Thus, these two Fano resonances can be independently regulated by different structural parameters. These findings can provide substantial theoretical basis in designing sensitive dual channel sensors.

## Data Availability

Not applicable.
